# Comprehensive cardiovascular MRI in hypertension: a UK single centre experience

**DOI:** 10.1186/1532-429X-16-S1-P236

**Published:** 2014-01-16

**Authors:** Punam Pabari, Klio Konstantinou, Nilesh Sutaria, Declan P O'Regan, Sanjeev Bhattacharyya, Niall G Keenan, Neil Chapman, Ben Ariff

**Affiliations:** 1 Imperial College NHS Trust, London, UK

## Background

MRI is a useful investigation in hypertensive patients, providing a non-invasive method for the assessment of the heart and potential secondary causes (including adrenal, aortic, renal and renovascular abnormalities). This is now more relevant with the introduction of therapies such as renal denervation in those with suitable anatomy. The ability to perform a single comprehensive assessment is both convenient for patients and potentially cost-effective. We describe the experience in the largest UK single-site series to date.

## Methods

193 patients (59% male and 41% female; median age 39 [range 17-83] years) were referred for hypertension screen MRI scans performed at St Mary's Hospital, London, between August 2011 and September 2013. Images were acquired on a GE 1.5 T HDXT Platform with an 8 channel cardiac array coil. For the hypertension screen protocol, cardiac and thoracic imaging was performed using standard SSFP cine imaging and supplemented with black blood fast spin imaging. T2 SSFP high resolution fat saturation imaging of the kidneys and adrenals with additional fast spoiled gradient echo sequences of the adrenal glands was used. Standard Gadovist contrast MRA of renal arteries was performed, with the option to include delayed myocardial enhancement. Volumetric analysis was performed using the GE Reportcard.

## Results

Of the 193 patients, 32% of the total had increased LV wall thickness. 19% had elevated LV mass. 2% had increased LV volumes. Dilated aortic roots (≥ 40 mm) was present in 9 patients (5%). 8 (4%) patients had renal artery stenosis (1 bilateral) and 12 (6%) had adrenal adenomas requiring biochemical characterisation; none had aortic coarctation or phaeochromocytoma. Significant incidental findings included 3 patients with breast lesions requiring further assessment, one with mediastinal and hilar lymphadenopathy (subsequently diagnosed as sarcoid), one with horseshoe kidney and one with hydronephrosis secondary to PUJ obstruction.

## Conclusions

In our experience MRI provides a rapid, comprehensive and convenient one-stop method for the identification of cardiac abnormalities and secondary causes of hypertension, particularly in young patients or those with resistant hypertension. In this large UK series potential secondary causes were identified in 20 (10%) patients, and other clinically significant abnormalities requiring follow-up in 15 (8%). MRI is a useful screening tool for selected hypertensive patients which can be integrated in the work up of patients considered for renal denervation.

## Funding

N/A.

**Figure 1:**
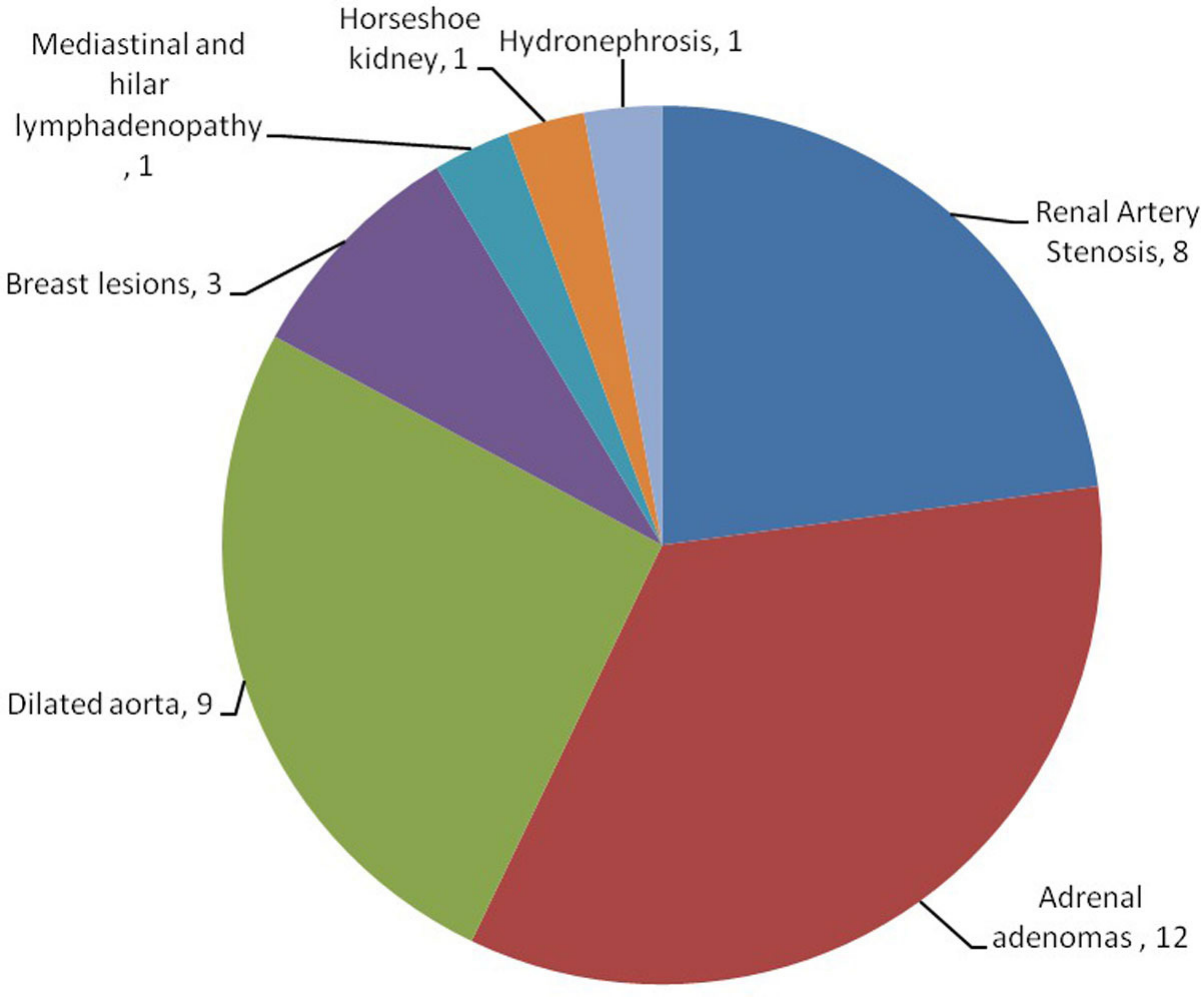
**35 patients (18%) had significant findings requiring further assessment as shown**.

